# Ecosystem Service Values in the Dongting Lake Eco-Economic Zone and the Synergistic Impact of Its Driving Factors

**DOI:** 10.3390/ijerph19053121

**Published:** 2022-03-07

**Authors:** Guangchao Li, Wei Chen, Xuepeng Zhang, Zhen Yang, Pengshuai Bi, Zhe Wang

**Affiliations:** 1 College of Geoscience and Surveying Engineering, China University of Mining & Technology, Beijing 100083, China; bqt1900205060@student.cumtb.edu.cn (G.L.); sqt1900205107@student.cumtb.edu.cn (X.Z.); zqt2000205116@student.cumtb.edu.cn (P.B.); sqt1900205103@student.cumtb.edu.cn (Z.W.); 2 College of Information Science and Engineering, Henan University of Technology, Zhengzhou 450001, China; zhenyang@haut.edu.cn

**Keywords:** ESVs, driving factors, Dongting Lake, XGBoost model, land use, GPP, SHAP

## Abstract

Ecosystem service values (ESVs) are crucial to ecological conservation and restoration, urban and rural planning, and sustainable development of land. Therefore, it is important to study ESVs and their driving factors in the Dongting Lake Eco-Economic Zone (Dongting Lake). This paper quantifies the changes in ESVs in the Dongting Lake using land use data from 2000, 2005, 2010 and 2018. The eXtreme Gradient Boosting (XGBoost) model is used to study the effects of individual driving factors and the synergistic effects of these driving factors on ESVs. Our analysis suggests that: (1) From 2000 to 2018, the largest dynamic degree values in the Dongting Lake are in unused land types, followed by construction lands and wetlands. The ESVs of the Dongting Lake show an increasing trend, with those of forestlands being the highest, accounting for approximately 44.65% of the total value. Among the ESVs functions, water containment, waste treatment, soil formation and protection, biodiversity conservation and climate regulation contribute the most to ESVs, with a combined contribution of 76.64% to 76.99%; (2) The integrated intensity of anthropogenic disturbance shows a U-shaped spatial distribution, decreasing from U1 to U3. The driving factors in descending order of importance are the human impact index, total primary productivity (GPP), slope, elevation, population, temperature, gross domestic product, precipitation and PM2.5; (3) When the GPP is low (GPP < 900), the SHAP (SHapley Additive exPlanation) value of the high human impact index is greater than zero, indicating that an increase in GPP increases the ESVs in the Dongting Lake. This study can provide technical support and a theoretical basis for ecological environmental protection and ecosystem management in the Dongting Lake.

## 1. Introduction

Land cover change is the main factor affecting terrestrial ecosystems, reflecting ecological, natural resource and land management developments due to natural and anthropogenic activities [1,2], and is key to understanding the interactions between anthropogenic factors and global changes [3]. Land use type changes can alter energy balances and biogeochemical cycles, further affecting the climate and ecosystem environments [4,5,6]. Ecosystem services (ESs) change along with global change [7,8], and ecosystem service values (ESVs) are assessments of the contributions of ecosystem service processes that consider the sustainability and rational allocation of ecological structure and function [9,10]. ESs refer to the direct and indirect contributions of ecosystems to human well-being and subsistence. Ecosystem valuation is an approach to assigning monetary values to an ecosystem and its key ecosystem goods and services, generally referred to as ESVs [11]. With the depletion of ecological resources and increasing human activities, ESVs assessments are a trend in the field of ecosystem research [12,13]. In the sustainable development of the ecological environment in China, the importance of ESVs assessment has been recognized in the long-term, stable development of the ecological environment and ecological restoration.

ESVs serve as a foundation for ecosystem conservation and planning, environmental policy decisions and land management [14,15,16,17,18]. ESVs is a quantitative assessment of the potential service capacity of ecosystems and a comprehensive evaluation of the interaction between the environment and human development, which usually provides a theoretical and practical basis in relevant decision-making and is a core indicator of whether a region can achieve sustainable development. With the in-depth understanding of ESVs, evaluation of ESVs has become an indispensable part of ecosystem investigations [19,20]. ESVs assessment methods include equivalence factors, productivity and biomass methods [21,22]. The coefficient method using land use/cover data is the most widely used ESVs assessment method [23,24,25]. Currently, the assessment of regional ecological service effects caused by land use changes from a value quantification perspective is widely used in ecology [26,27]. ESVs have been applied in different ecosystem types, such as watersheds [28,29,30], cities [22,31,32,33], mountains [34,35], ecologically fragile areas [36,37] and wetlands [38,39].

Most of the above studies have estimated regional ESVs and analyzed their dynamics; however, the analysis of the driving factors of ESVs contributes to the understanding of an ecosystem’s environmental protection and formation mechanisms [40]. Understanding the changes in ESVs and their driving factors is the basis for regulating a positive response of ecosystems to the driving factors [41]. There are also many studies on the driving factors of ESVs [42], and the main factor is land use change, which is mainly influenced by anthropogenic factors [43,44]. Ecosystem driving factors mainly include land use, natural and socioeconomic factors [45]. There are also studies that point to indirect driving factors of ESVs, such as economic [46], topography [47,48], urbanization [49,50], climate [51,52,53], population [54] and social development [55,56] factors. The change in ESVs is caused by the interaction of multiple driving factors. In this complex process, it is necessary to comprehensively analyze the synergy between different driving factors to understand the synergistic effect of driving factors on changes in ESVs [45,57]. Therefore, a comprehensive analysis of the synergistic impact of driving factors on ESVs provides a reference for understanding and controlling trade-offs between ESVs and their driving factors.

In 1994, Dongting Lake was identified as a national nature reserve by the State Council and was listed as an extremely important biodiversity reserve in China. The Dongting Lake undertakes important functions, such as maintaining a regional ecological balance, guaranteeing water and ecological security in the Yangtze River Basin, and safeguarding national food security. Research on the supply–demand balance of various ecological services plays an important role in coordinating regional interest relations, promoting urban-rural and regional coordination and common prosperity, ecological restoration and overall social development. The State Council officially approved the Dongting Lake plan in April 2014. Therefore, studying the changes in ESVs and the synergistic effects of their drivers in the Dongting Lake can support the ecological control and sustainable development of the Dongting Lake.

Previous studies were more based on linear methods to study the driving factors of ESVs, and only studied the impact of a single driving factor on ESVs. However, there are nonlinear and complex synergistic relationships between ESVs and driving factor. Therefore, the significance of this study is mainly to use the XGBoost model (nonlinear method) to quantify the importance of a single driving factor on ESVs, and to study the effect of synergistic effects of different driving factor on ESVs. The main research purposes of this paper are to (1) analyze the land use changes in the Dongting Lake in 2000, 2005, 2010 and 2018; (2) study the system composition of ESVs of the Dongting Lake; (3) analyze the spatial distribution and change trend of ESVs of the Dongting Lake in 2000, 2005, 2010 and 2018; and (4) quantify the importance of individual driving factors and the impact of synergies between drivers on the ESVs.

## 2. Data and Methods

### 2.1. Data

**Figure 1:**
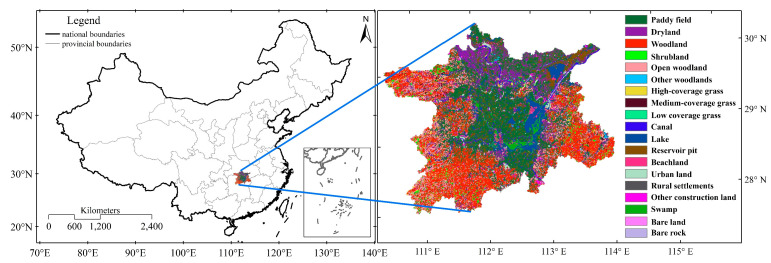
The geographic location and spatiotemporal distribution of land use types in the Dongting Lake in 2018.

In this study, remote sensing data for the years 2000, 2005, 2010 and 2018 were selected, in which land use data, population (POP), gross domestic product (GDP), elevation (DEM) and slope (Slope) data were obtained from https://www.resdc.cn/ (accessed on 19 February 2022) The spatial resolution of the land use data was 1 km and included 19 land use types (Figure 1). In this study, 19 land use types were reclassified into seven categories, including cultivated land (CL), forestland (WO), grassland (GL), water area (WA), construction land (CO), unused land (UL) and wetland (WL). In this paper, unused land is defined as sandy land, Gobi, saline–alkali land, swampy land, bare land, bare rock texture and other unused land, including alpine desert, tundra, etc. Spatialization of the POP and GDP data was achieved based on a multifactor weight assignment method and multiple factors closely related to population with a resolution of 1 km. The elevation and slope data were based on the latest SRTM V4.1 (National Aeronautics and Space Administration, Washington, DC, USA) data that were resampled to produce a 1 km resolution dataset. The spatial resolution of the gross primary productivity (GPP) of the vegetation was 0.05° (http://www.geodata.cn/) (accessed on 27 December 2021) [58]. Precipitation (Pre) and temperature (Temp) data were datasets obtained by Peng, et al. [59] based on a bilinear interpolation method with a spatial resolution of 0.5’. PM2.5 (PM) data were obtained from https://sites.wustl.edu/acag/ (accessed on 19 February 2022) with a spatial resolution of 0.01°. The driving factors used in this paper are all averaged in counties, and then calculated and analyzed.

### 2.2. Study Area

The Dongting Lake includes four prefecture-level cities and one district in Hunan Province and Hubei Province. The Dongting Lake is located at 27°98′–30°23′ N, 110°20′–114°14′ E (north latitude). The area of the whole district is 60,500 km^2^, with a total population of 22 million residents. The main topography of the Dongting Lake includes vast alluvial plains, lakes and water networks, and the surrounding mountains and hills have a complex topography that also includes a large area of wetlands. Dongting Lake is an important base for commercial food, aquaculture and farming in China. The annual mean temperature range of the Dongting Lake is 16.4–17.0 °C, the frost-free period is 260–280 d, and the annual mean precipitation range is 1200–1550 mm. The geographic location and spatiotemporal distribution of land use types of the Dongting Lake in 2018 are shown in Figure 1.

Dongting Lake is the second largest freshwater lake in China. The lake area is known as the “land of fish and rice”. It is an important commercial grain base, aquatic product and breeding base in China. The Dongting Lake ecosystem can play a role in regulating runoff, storing floods and preventing droughts, conserving water sources, purifying water quality, purifying air, regulating climate, protecting organisms and enriching species. According to the statistics of biodiversity in Dongting Lake wetland ecosystem, there are 170 families, 637 genera and 1428 species of plants, 11 orders, 22 families and 119 species of fish, 16 orders, 43 families and 216 species of birds, eight orders, 13 families and 22 species of mammals and 27 species of amphibians and reptiles [60]. It is also an important habitat for the rare species of finless porpoise in the Yangtze River, and is listed as one of the 200 important ecological areas in the world by the World Wildlife Fund. Therefore, the protection and sustainable utilization of the Dongting Lake ecosystem is of great significance for ensuring the steady development of the regional economy and society and improving the ecosystem environment.

### 2.3. Methods

#### 2.3.1. Land Use Changes

In this paper, a single land use type dynamic degree was used to measure the magnitude of land use type changes in the Dongting Lake, which is calculated as follows:

(1) $$K=\frac{L_{b}-L_{a}}{L_{a}}\times \frac{1}{H}\times 100\%$$

where K denotes the land type dynamic degree, $L_{a}$ and $L_{b}$ are the areas of a land use type in 2000 and 2018, respectively, and H denotes the study time period.

#### 2.3.2. Ecosystem Service Values

This study used the equivalence factor method to calculate the ESVs of the Dongting Lake [61], defining the standard unit of an ESVs equivalence factor as the economic value of the national average natural grain yield per year for a 1 hm^2^ farmland. The calculation formula was:

(2) $$V=R_{1}\times V_{1}+R_{2}\times V_{2}+R_{3}\times V_{3}$$

where V represents the ESVs of 1 standard equivalent factor (yuan/hm^2^) and $R_{1}$, $R_{2}$ and $R_{3}$ denote the proportion of wheat, corn and rice, respectively, in the total area of the three crops in 2010. $V_{1}$, $V_{2}$ and $V_{3}$ denote the net profit per unit area (yuan/hm^2^) of wheat, corn and rice, respectively, in 2010. The value of V was 1793.88 yuan/hm^2^ according to the 2011 China Statistical Yearbook, 2011 National Compilation of Agricultural Product Cost–Benefit Information and Equation (2).

From this, the value of the ecological services of the Dongting Lake was calculated with the following equations:

(3) $$VL_{ti}=EL_{ti}\times E_{b}$$

where $VL_{ti}$ denotes the ESVs coefficient of the *i*-th land use type, $EL_{ti}$ denotes the equivalent value of the *t*-th ecosystem service to the *i*-th land use type, and $E_{b}$ denotes 1 standard equivalent ecosystem service value.

(4) $$ESVs=\sum_{i=n}^{n}\left(B_{i}\times \sum_{t=1}^{k}VL_{ti}\right)$$

where ESVs denote ecosystem service values and $B_{i}$ denotes the area of the *i*-th land use type. In this paper, construction land was assigned an ESVs of 0 in reference to previous studies. Table 1 shows the ESVs for each land use type per unit area of the Dongting Lake.

#### 2.3.3. Human Impact Index

The human impact index (HAI) represents the impact of human activities on land cover types and landscape changes, and the interrelationship between ESVs and the intensity of anthropogenic disturbance in the study area can be analyzed using the HAI, which is calculated as follows:

(5) $$HAI=\sum_{i=1}^{n}\frac{B_{i}P_{i}}{TA}$$

where $B_{i}$ denotes the area of the *i*-th land use type, TA is the total land use area of the Dongting Lake area, and $P_{i}$ is the intensity coefficient of anthropogenic impact. In reference to previous studies, this study uses the Delphi method to assign a $P_{i}$ value of 0.67 for farmland, 0.13 for forestland, 0.1 for grassland, 0.12 for water, 0.96 for construction land, 0.05 for unused land and 0.15 for wetland land cover types [62].

#### 2.3.4. Correlation Analysis

1. XGBoost algorithm

XGBoost is a Boosting algorithm belonging to an ensemble learning model. With the most efficient performance on supervised learning tasks such as classification, regression, and ranking, the algorithm has become a tool of choice for machine learning, primarily due to its excellent prediction performance, highly optimized multi-core processing and distributed machine implementation, and the ability to handle sparse data. The XGBoost algorithm is a gradient boosting, gradient lifting method that uses an addition model and a forward distribution algorithm to gradually approach the optimal result. Moreover, the XGBoost algorithm simultaneously prevents model overfitting by introducing a regularization term (a measure of tree model complexity) in the objective function [63], which is calculated as follows:

(6) $${\hat{G}}_{j}^{N}=\sum_{k=1}^{N}\phi_{k}(x_{i})={\hat{G}}_{j}^{N-1}+\phi_{N}(x_{j})$$

where ${\hat{G}}_{j}^{N-1}$ is the generated tree, $\phi_{N}(x_{j})$ is the newly created tree model and *N* is the total number of tree models. In this study, the ESVs of the study area were used as dependent variables, and various natural and social factors were used as independent variables. All variables were input into the XGBoost model, and the model was cleaned, normalized and trained on the input data to obtain the determined relationship model.

2. SHAP method

Machine learning has great research significance and wide application value for time series prediction, but its application in real-world tasks is severely limited due to the lack of interpretability. Interpretability is very important in the ecological field, and research on constructing interpretable deep learning models for ESVs can provide complete logic for corresponding decision-making. To explain the extent to which different drivers contribute to the machine learning prediction results, the SHAP method was applied to the predictions of XGBoost [64]. The SHAP method belongs to the post-event interpretation framework, which estimates the contribution of each driving factor through the Shapley value, which is the core of SHAP. Compared with traditional feature importance methods, SHAP has better consistency and can calculate the impact of each driver on the prediction results, helping researchers interpret machine learning predictions. Therefore, it can be used to explain the contribution of each driver to the prediction, adding transparency and facilitating the analysis of the degree of response of ESVs to different drivers. The SHAP formula is as follows:

(7) $$\hat{\varphi_{i}}=\frac{1}{N}\sum_{n=1}^{N}\left(\hat{f}(y_{+i}^{h})-\hat{f}(y_{-i}^{h})\right)$$

where $\hat{f}(y_{+i}^{h})$ represents the prediction of *y*.

Shapley values are the only way to explain feature importance, satisfying the mathematical properties of local accuracy and consistency, while TreeExplainer is an interpreter specialized in interpreting tree models, activating Shapley values for feature attribution through tree ensemble and additive methods. TreeSHAP can be used in the gradient enhancement model [65]. Compared with classical algorithms, TreeSHAP has many optimizations, and can provide rich visualization of each driver compared to classical feature importance and partial dependency graphs. The formula is as follows:

(8) $$\varphi_{m,n}=\sum_{R\subseteq P\left\{m,n\right\}}\frac{\left|R\right|!(N-\left|R\right|-2!)}{2(N-1)!}\varepsilon_{mn}(R)$$

where m≠n, $\varepsilon_{mn}(R)=g_{x}(R\cup \left\{m,n\right\})-g_{x}(R\cup \left\{m\right\})-g_{x}(R\cup \left\{n\right\})+g_{x}(R)$, *N* is the number of features and *R* represents all feature subsets.

## 3. Results

### 3.1. Land Use Changes

**Figure 2:**
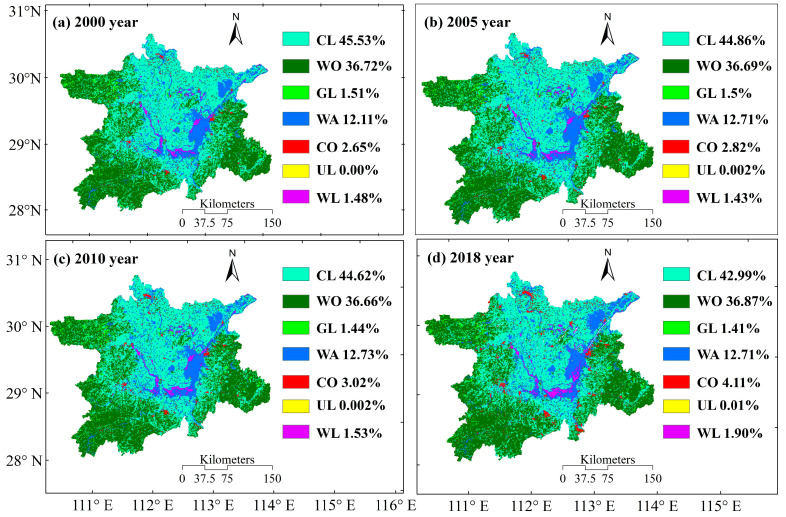
Spatial distribution and percentage of land use in the Dongting Lake.

Land use in the Dongting Lake from 2000, 2005, 2010 and 2018 was mainly dominated by croplands and forestlands, and the combined area of both land use types accounted for more than 79% of the total area, with a very small proportion of the area representing unused land (Figure 2). During the 2000–2018 period, the area shared by all land use types showed an increasing trend, except for a decrease in the area shared by cropland and grassland. Among these land use types, cultivated land and construction land changed the most, with a reduction in area of 2.54% and an increase in area of 1.46%, respectively.

**Figure 3:**
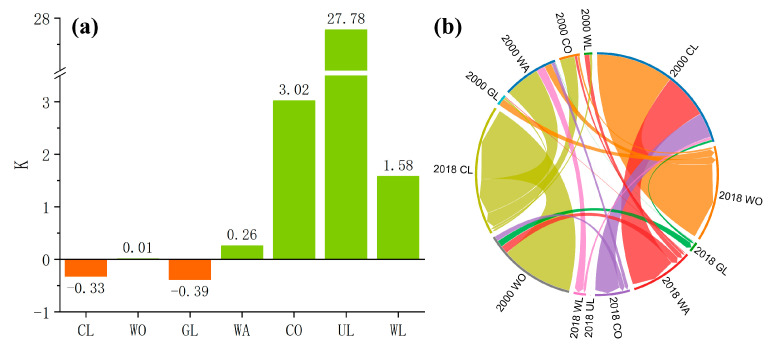
Dynamic degree (**a**) and mutual transformation (**b**) of land use changes in the Dongting Lake in 2000 and 2018.

The changes in single land use dynamic degree and interconversion in the Dongting Lake in 2000 and 2018 show that the largest value of the dynamic degree in unused land was due to the expansion of this land use type from 0 km^2^ to 6 km^2^ (Figure 3). For the convenience of calculation, we assumed that the area of the unused land use type in 2000 was 1 km^2^ (1 km^2^ in 2005 and 2010). Construction lands and wetlands had the second largest values, with dynamic degrees of 3.02 and 1.58, respectively. The dynamic degree of the other four land use types was relatively small, among which the dynamic degrees of cultivated land and grassland were negative, which are −0.33 and −0.39, respectively. The dynamic degree of land use is closely related to the intensity of human activities, such as the encroachment of cultivated land, the expansion of construction land, and the implementation of ecological restoration projects such as returning cultivated land to forestland and grassland, resulting in changes in the dynamic degree of land use types in different cities in Dongting Lake (Figure 3a). From 2000 to 2018, the area of construction land, water area, wetland, forest land and unused land increased, mainly from the conversion of cultivated land, which is also the reason for the decrease of cultivated land area. The increase in wetland area is mainly due to the conversion of water area, while the decrease in grassland area is mainly due to the conversion of grassland to forestland. The area of cultivated land converted to forest land was the largest, followed by the area of forest land converted to cultivated land; except for cultivated land, water area and wetland, other land use types were not converted to unused land use types (Figure 3b).

### 3.2. Value of Each Service Type in Different Ecosystems

Structure determines function, and analysis of the value changes at the level of the land use structure is important for an in-depth study of the causes of changes in regional ESVs. ESVs for each service function of the different ecosystems in the Dongting Lake in 2000, 2005, 2010 and 2018 are shown in Table 2. Forestlands had the largest ESVs, accounting for approximately 44.65% of the total value, followed by watersheds (32.13%), croplands (17.11%) and wetlands (5.52%), and grasslands, wastelands and construction lands had low percentages. There was an overall increasing trend in ESVs in the Dongting Lake during 2000–2018. The ESVs of wetland and water body land types increased, reaching CNY 2.83 billion and CNY 2.81 billion, respectively, followed by forestland, where the ESVs increased by CNY 0.06 billion. Both cropland and grassland ESVs decreased, with cropland ESVs decreasing the most (CNY 2.01 billion). In terms of the ESVs growth rate, the wetland growth rate was the highest (28.28%), and the grassland growth rate was the lowest (−7.00%).

In terms of the functional composition of ESVs, the ESVs of the Dongting Lake showed an upward trend from 2000 to 2018, and the ESVs increased by CNY 3.62 billion during this time period. The watershed had the highest value for water conservation and waste treatment, with the sum of both remaining above CNY 50 billion, reaching its highest value in 2010 (CNY 53.21 billion). This was followed by soil formation and protection and gas regulation in forests, both of which remained above CNY 29 billion combined, reaching a maximum value in 2019 (CNY 29.47 billion). The composition proportion of various functions changed little. Among these functions, water conservation, waste treatment, soil formation and protection, biodiversity protection and climate regulation contributed the most to the ESVs, with a total contribution rate of 76.64~76.99%, followed by gas regulation, entertainment culture, raw materials and food production. The largest contribution was from water connotation (23.39~23.86%), and the smallest was from food production (2.69~2.89%). In addition to gas regulation, soil formation and protection, food production and raw materials, the ESVs of other functions increased, among which the increase in water conservation was the most obvious (1.77 billion yuan).

### 3.3. Spatial Distribution of ESVs

**Figure 4:**
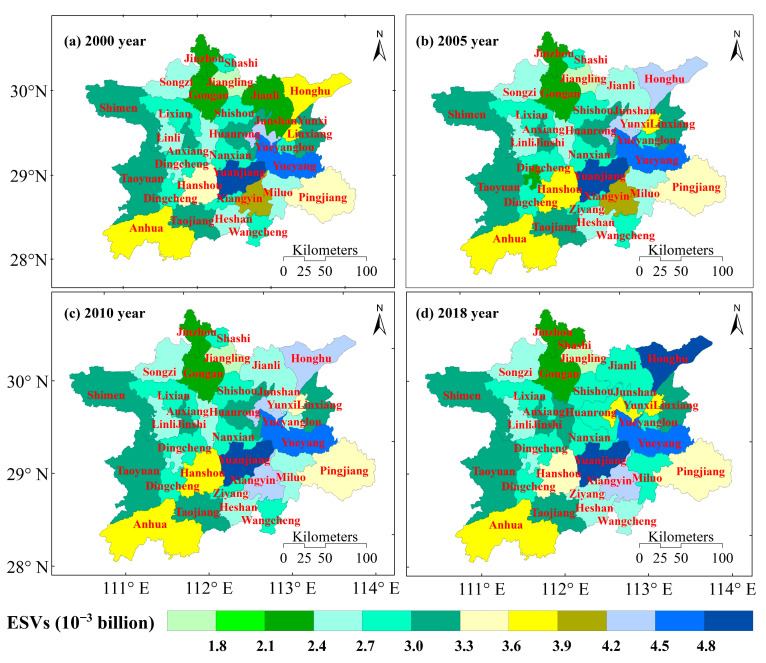
Spatial distribution of ESVs per county (city, district) in the Dongting Lake.

There was little difference in the spatial distribution of ESVs in Dongting Lake counties (cities and districts) between 2000, 2005, 2010 and 2018 (Figure 4). From 2000 to 2018, the counties with significantly reduced ESVs included Junshan (CNY 0.67 × 10^−3^ billion), Shashi (CNY 0.43 × 10^−3^ billion), Wangcheng (CNY 0.36 × 10^−3^ billion), Huanrong (CNY 0.32 × 10^−3^ billion) and Ziyang (CNY 0.18 × 10^−3^ billion). Counties with significant increases in ESVs included Honghu (CNY 0.93 × 10^−3^ billion), Anxiang (CNY 0.62 × 10^−3^ billion), Jianli (CNY 0.43 × 10^−3^ billion), Yuanjiang (CNY 0.34 × 10^−3^ billion), Xiangyin (CNY 0.26 × 10^−3^ billion), and Miluo (CNY 0.21 × 10^−3^ billion). The ESVs in other counties (cities and districts) did not change significantly. The high value areas of ESVs in the Dongting Lake were concentrated in the areas with more water area and forestland land use types, that is, areas with less interference from human activities, including Yuanjiang, Honghu, Yueyang, Anhua and Hanshou counties. The low value areas of ESVs in the Dongting Lake were mainly concentrated in areas with more arable land area, i.e., areas with more anthropogenic disturbance, including Jinzhou, Gongan and Jiangling counties.

### 3.4. Machine Learning Analysis of Driving Factors

#### 3.4.1. Human Impact Index

**Figure 5:**
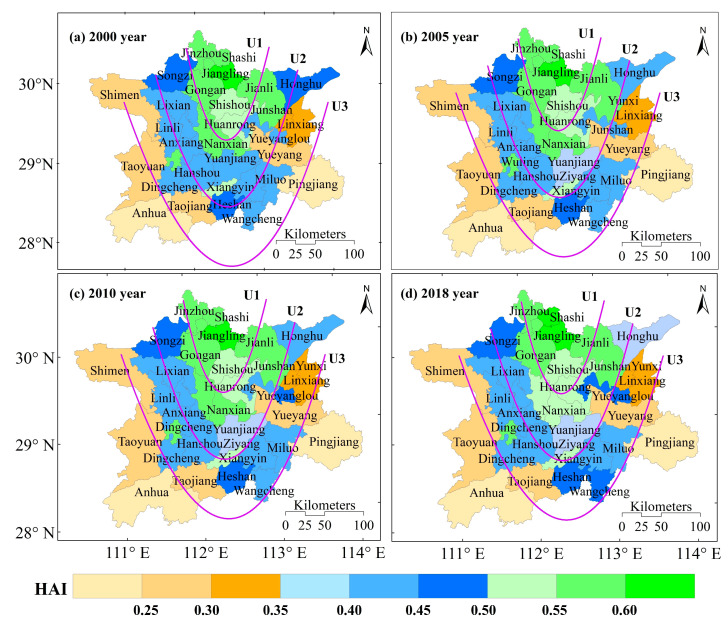
Spatial distribution of the HAI in Dongting Lake counties (cities and districts).

With improvements in social productivity, human activities have an important impact on ecosystem patterns. To quantitatively analyze the impact of anthropogenic disturbance on the changes in ESVs in the Dongting Lake, we assessed the human impact index in 2000, 2005, 2010 and 2018, using counties as units (Figure 5). The human impact index in the Dongting Lake showed a U-shaped spatial distribution, decreasing from U1 to U3. In areas with a high anthropogenic impact intensity (HAI > 0.5), the intensity of the anthropogenic impact in Shashi increased, the intensity of the anthropogenic impact in Anxiang and Nanxian decreased, and the other areas were basically unchanged. In areas with a medium anthropogenic impact intensity (0.35 ≤ HAI ≤ 0.5), the anthropogenic impact intensity increased in Wangcheng, Junshanh and Yueyanglou, decreased in Honghu, and remained basically unchanged in other areas. In areas with a low anthropogenic impact intensity (HAI < 0.35), the intensity of the anthropogenic impact remained basically unchanged. This study showed that the areas of high disturbance intensity, which were dominated by cropland and built-up land use types, were located in areas with low ESVs.

#### 3.4.2. Driving Factor Analysis

**Figure 6:**
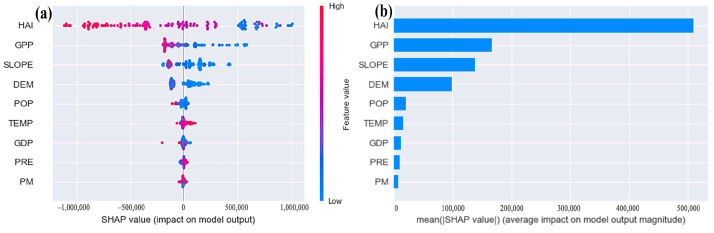
SHAP summary (**a**) and relative importance of each feature (**b**) of the XGBoost model of the Dongting Lake in 2000, 2005, 2010 and 2018.

Figure 6 shows the summary plot of the XGBoost model SHAP and the relative importance of each driving factor in the Dongting Lake in 2000, 2005, 2010 and 2018. In this model, the driving factors were the HAI, GPP, Slope, DEM, POP, Temp, GDP, Pre and PM in descending order of importance. This indicated that the combined human impact index was the most important driver, followed by GPP, and the ESVs of Dongting Lake may increase with an increasing GPP. In contrast, PM had essentially no effect on the ecological and economic changes in Dongting Lake.

**Figure 7:**
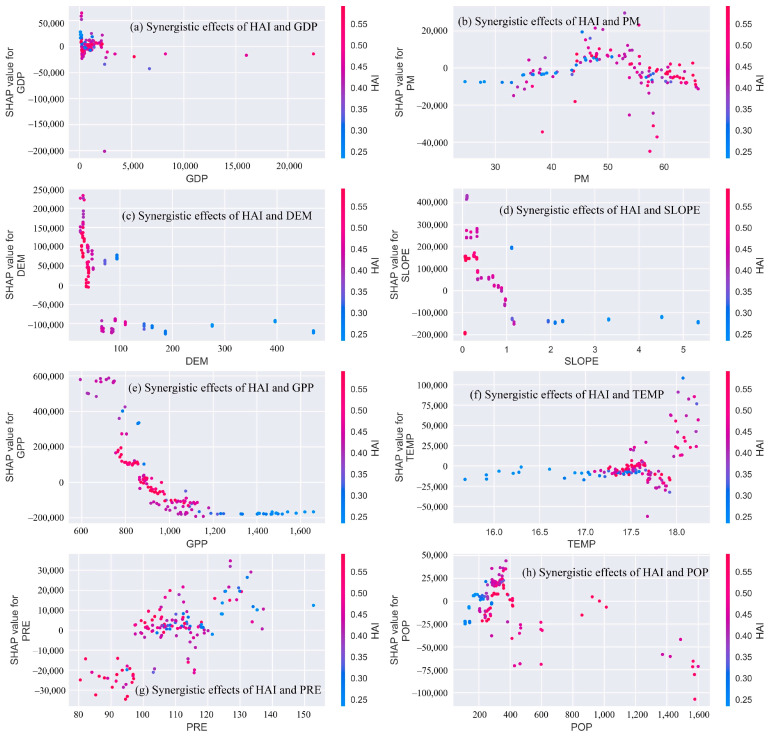
Shape dependence plot of the importance of driving factors in the Dongting Lake in 2000, 2005, 2010 and 2018. The *x*-axis represents the driving factor values, and the *y*-axis represents the SHAP values. The red to blue bars on the right sides of the graphs indicate high to low values of the HAI, respectively.

A SHAP dependency plot enables us to examine the relationship between driving factors and ecological values, and Figure 7 shows the SHAP dependency plot of the HAI and other driving factors in the Dongting Lake. The SHAP dependency plot allows the SHAP values of the HAI to be compared with other driving factors and shows the influence of all driving factors on the ecological value of the Dongting Lake and the synergistic effect of the HAI with other driving factors. The SHAP value indicates the negative and positive contribution of driving factors to the model output variable and shows the contribution of each driving factor to the ecological value of the Dongting Lake. For example, Figure 7e represents the change in the ESVs as the GPP changes, and the vertical color bar indicates the synergistic effect of a single GPP value with the HAI. We can examine the effect of the GPP on the ecological value as the HAI rises from 0.25 to 0.55. The red to blue bars indicate high to low HAI values, respectively, indicating that an increase in the HAI increases the volatility of the ESVs. When the GPP is low (GPP < 900), the SHAP value of the high HAI is greater than zero, indicating that an increase in the GPP of the Dongting Lake increases the ESVs. Figure 7c represents the effects of the DEM and HAI on ESVs changes, indicating that when the SHAP value of the DEM is greater than zero, the increased DEM will lead to an increase in the ESVs; moreover, high values of the DEM and low values of the HAI indicate that the ESVs tends to be lower.

## 4. Discussion

### 4.1. Spatial Variation of ESVs

Rational planning for land use change can have positive implications for ecosystem conservation and sustainable development in the Dongting Lake. This study quantitatively analyzes the change in ESVs of the Dongting Lake caused by land use changes in 2000, 2005, 2010 and 2018. The overall land use of the Dongting Lake was relatively stable, indicating a stability in ESVs during this study period. However, the complex, nonlinear changes in the ESVs, as well as the different evaluation methods, make the ESVs results somewhat different [40]. Both construction land and forested land area in the Dongting Lake increased during 2000–2018, which is in line with the trend of China’s forest resource growth and urbanization development, and is related to the forest protection and expansion plan implemented in China [66]. Water bodies, forests and wetlands dominated the changes in ESVs [41,67], while increases in built-up land and unused land areas dominated the decrease in ESVs [68]. The total coverage area of forests, water bodies and wetlands accounted for more than 50% of the total area of the Dongting Lake, and these land cover types were the main components of the ESVs of the Dongting Lake. Water conservation [69] and waste treatment [70] were the main regulating functions of the Dongting Lake because the Dongting Lake has large water and wetland coverage areas that are of great significance to the hydrological functioning of the Dongting Lake.

To protect Dongting Lake’s ecosystem and its endangered rare species, strengthen environmental planning and biodiversity monitoring in the surrounding area. In the planning of functional areas, it is necessary to scientifically plan biodiversity conservation areas, especially to define core protected areas and peripheral protected areas. In areas with high ESVs in Dongting Lake, strengthen the protection of forest land and waters, comprehensively implement projects such as cleaning up the core area of protected areas and ecological restoration, and establish projects such as returning farmland to lakes, returning farmland to wetlands, wetland ecological restoration and controlling the impact of human activities on the disturbance of biodiversity in the region. In the areas with low ESVs in Dongting Lake, establish the project of returning farmland to forest, strengthen the protection of water sources, reduce the disturbance of human activities to the ecosystem and improve the habitat volume of the entire ecosystem type. We must adopt different protection and development models according to local conditions in order to maintain the structural stability and service functions of the Dongting Lake ecosystem.

### 4.2. Driving Factor Analysis

Analysis of the relationship between ESVs and their driving factors can provide an important basis for scientific ESVs research, ecological protection and restoration, urban and rural planning and sustainable development of land [57,71]. Many studies have identified anthropogenic influences as the main driving factors of ESVs [41,72,73], and the results of this study are consistent with these studies. The GPP represents the total amount of organic matter produced by vegetation and the initial material and energy of ecosystems [71,74]. Therefore, it is logical that it was the second most important factor influencing the ESVs. The heterogeneous environment created by slopes in certain areas affects environmental changes in their ecosystems and thus changes the land cover types. Slope data are generated from elevation, and the influence of these geographic features on ESVs is also important [40,45,75]. The effects of temperature and precipitation on ESVs vary with geographic location [76]. In addition, population density and economic factors also have important effects on ESVs [40]. This is the reason why eight influential factors were selected to analyze the driving factors of ESVs in this paper.

ESVs are complex processes in which many influencing factors act synergistically, and only analyzing the effects of individual driving factors on ESVs cannot reveal the contributions of driver synergies to the ESVs. Therefore, it is important to quantify the effect of synergistic interactions among driving factors on ESVs [40,77]. For example, Liu, et al. [45] concluded that the higher the population density in the critical value range, the higher the value of ESVs in areas with a high richness of land cover types. Wang, et al. [71] showed that the interaction between the NPP and soil conservation increased when the slope decreased, indicating that the synergy of these driving factors can be increased by changing the driving factors of the ESVs. Pan, et al. [41] showed that the differences in the spatial distribution of ESVs in the study area were caused by the synergistic effects of anthropogenic factors, natural conditions, and landscape patterns. This paper quantitatively analyzes the effects of the HAI in synergy with other driving factors on ESVs and lays the foundation for exploring the differences in the spatial distribution of ESVs and their complex relationships with their driving factors.

### 4.3. Limitations and Future Work

The study of the factors influencing ESVs currently needs further improvement. This paper analyzes the importance of nine driving factors (HAI, GPP, Slope, DEM, POP, Temp, GDP, Pre and PM) on ESVs; however, ESVs also respond to other influencing factors such as oxygen release [57], the normalized difference vegetation index [41] and soil conditions [40]. Therefore, further refinement is still needed to comprehensively consider the effects of driving factors on ESVs in future studies to fully reflect the values of the ESVs. In addition, the data used in this paper may have had accuracy uncertainties of their own due to the limitations and effects of the raw data and other conditions. Therefore, data with a higher inversion accuracy can be used to improve the analysis results in future studies.

Furthermore, this study only analyzed the effect of the HAI in synergy with other driving factors on ESVs and did not investigate the effects of other driving factors in synergy with each other on the ESVs. In future work, the effects on ESVs of the synergies of these other driving factors with each other will be developed. In addition, although this study analyzed the impact of driver synergies on ESVs, drivers such as other natural conditions and policies in the different study areas could not be quantified, and the impacts of these drivers were not analyzed. In future research, some policies and other natural conditions can be included as impact factor indicators.

## 5. Conclusions

In this paper, we studied the impact of land use changes on ESVs in the rapidly-developing Dongting Lake region in 2000, 2005, 2010 and 2018, and quantified the impacts of individual driving factors and their synergies on the ESVs using XGBoost models. The conclusions were as follows:

(1) The largest changes in land use areas of the Dongting Lake from 2000 to 2018 were in cropland (2.54% decrease) and construction land (plus 1.46%) and the largest values of dynamic degree were in unused land use types, followed by construction land and wetlands.
(2) The ESVs of the Dongting Lake showed an increasing trend, and the forestland ESVs were the highest, accounting for approximately 44.65% of the total value. Among the ESVs functions, water containment, waste treatment, soil formation and protection, biodiversity conservation and climate regulation contributed the most to the ESVs, with a combined contribution of 76.64% to 76.99%.
(3) The integrated intensity of anthropogenic disturbance showed a U-shaped spatial distribution, decreasing from U1 to U3. The driving factors in descending order of importance were HAI, GPP, Slope, DEM, POP, Temp, GDP, Pre and PM.
(4) When the GPP was low (GPP < 900), the SHAP value of the high HAI was greater than zero, indicating that an increase in the GPP of the Dongting Lake increases the ESVs. When the SHAP value of the DEM was greater than zero, an increase in the DEM led to an increase in the ESVs. Moreover, the high DEM values and low HAI values indicated that the ESVs tended to be lower.

## Figures and Tables

**Table 1:** ESVs per unit area by land use type in the Dongting Lake (Unit: Yuan/hm^2^).

Primary Type	Secondary Type	Abbreviation	CL	WO	GL	WA	UL	WL
Provision	Food production	FP	1793.88	179.39	538.16	179.39	17.94	538.16
	Raw material	RM	179.39	4664.08	89.69	17.94	0.00	125.57
Regulation	Gas regulation	GR	896.94	6278.58	1435.10	0.00	0.00	3228.98
	Climate regulation	CR	1596.55	4843.47	1614.49	825.18	0.00	30,675.33
	Water supply	WS	1076.33	5740.41	1435.10	36,559.25	53.82	27,805.12
	Waste treatment	WD	2941.96	2349.98	2349.98	32,612.71	17.94	32,612.71
Support	Soil formation and retention	SFR	2619.06	6996.13	3498.06	17.94	35.88	3067.53
	Biodiversity protection	BD	1273.65	5848.04	1955.33	4466.76	609.92	4484.70
Culture	Recreation and culture	EC	17.94	2296.16	71.76	7785.43	17.94	9956.03
Total			12,395.70	39,196.25	12,987.68	82,464.60	753.43	112,494.13

**Table 2:** The ESVs of various service functions of different ecosystems of the Dongting Lake in 2000, 2005, 2010 and 2018 (billion yuan).

LULC	Year	GR	CR	WS	SFR	WD	BD	FP	RM	EC
CL	2000	2.467	4.392	2.961	7.204	8.092	3.503	4.934	0.493	0.049
	2005	2.431	4.327	2.917	7.098	7.973	3.452	4.861	0.486	0.049
	2010	2.418	4.304	2.902	7.060	7.931	3.434	4.836	0.484	0.048
	2018	2.322	4.133	2.786	6.779	7.615	3.297	4.643	0.464	0.046
WO	2000	13.930	10.746	12.736	15.522	5.214	12.975	0.398	10.348	5.095
	2005	13.916	10.735	12.723	15.506	5.208	12.962	0.398	10.337	5.089
	2010	13.907	10.728	12.715	15.496	5.205	12.953	0.397	10.331	5.086
	2018	13.940	10.754	12.745	15.534	5.218	12.984	0.398	10.356	5.098
GL	2000	0.131	0.148	0.131	0.320	0.215	0.179	0.049	0.008	0.007
	2005	0.130	0.146	0.130	0.317	0.213	0.177	0.049	0.008	0.006
	2010	0.125	0.140	0.125	0.304	0.204	0.170	0.047	0.008	0.006
	2018	0.122	0.137	0.122	0.297	0.200	0.166	0.046	0.008	0.006
WA	2000	0.000	0.604	26.743	0.013	23.856	3.267	0.131	0.013	5.695
	2005	0.000	0.634	28.081	0.014	25.050	3.431	0.138	0.014	5.980
	2010	0.000	0.635	28.121	0.014	25.086	3.436	0.138	0.014	5.989
	2018	0.000	0.632	27.990	0.014	24.968	3.420	0.137	0.014	5.961
WL	2000	0.288	2.733	2.477	0.273	2.906	0.400	0.048	0.011	0.887
	2005	0.279	2.647	2.400	0.265	2.814	0.387	0.046	0.011	0.859
	2010	0.299	2.837	2.572	0.284	3.017	0.415	0.050	0.012	0.921
	2018	0.369	3.506	3.178	0.351	3.728	0.513	0.062	0.014	1.138

## Data Availability

Data sharing not applicable.
